# The Relationship between Specific Cognitive Domains, Fear of Falling, and Falls in People with Multiple Sclerosis

**DOI:** 10.1155/2014/281760

**Published:** 2014-07-24

**Authors:** Alon Kalron

**Affiliations:** ^1^Department of Physical Therapy, Sackler Faculty of Medicine, Tel Aviv University, 46379 Tel Aviv, Israel; ^2^Physical Rehabilitation Research Unit, Multiple Sclerosis Center, Sheba Medical Center, Tel Hashomer, 52621 Ramat Gan, Israel

## Abstract

The primary aim was to examine the relationship between seven definite aspects of cognition measured by a computerized cognitive testing tool on the history falls in people with mild to moderate MS (PwMS). Secondary aims focused on whether cognition performance is correlated to fear of falling, walking velocity, and a patient-rated measure of walking ability. One hundred and one PwMS were included in the study analysis. Fifty-two had a history of at least one fall during the past year. Outcome measures included a computerized cognitive test battery designed to evaluate multiple cognitive domains, gait speed, and self-reported questionnaires; 12-item MS walking scale (MSWS-12); and Falls Efficacy Scale International. Significant differences between fallers and nonfallers were exhibited in attention and verbal function, scoring 7.5% (*P* = 0.013) and 6.2% (*P* = 0.05), respectively, below the parallel scores of the nonfallers. Attention was the only cognitive component significantly correlated with the MSWS-12 self-reported questionnaire. Fear of falling was significantly correlated with 6 (out of 7) definite cognitive variables. The present findings support the concept that when evaluating and attempting to reduce fall risk, emphasis should be placed not only on traditional fall risk factors like muscle strength and motor function, but also on cognitive function.

## 1. Introduction

Falls in people with multiple sclerosis (PwMS) are a serious health concern. Studies have confirmed that more than 50% of falling incidents occur over a 6-month period [1–3]. PwMS have an increased risk for fracture compared with non-MS age-matched populations and, in particular, an increased risk of fragility fractures. Hence, this population routinely requires medical attention for fall-related injuries [3–5]. Furthermore, the effect of a fall extends past the actual adverse event as a fall can result in curtailment of activity, physiological deconditioning, and institutionalization [6].

Various fall risk factors have been documented in the MS population: poor postural control [7–9], disability status [2, 6, 9–11], fear of falling [1, 2, 7], gait difficulties [2, 8–10], sensory impairments [2, 10], and the use of an assistive device [2, 3, 6, 8, 9, 11]. However, according to authors of a meta-analysis, these findings should be interpreted with caution due to the relatively small number of studies and the variable methodological quality of the studies [12].

High prevalence of falls among PwMS, despite relatively intact motor function, underscores the belief that falls are not only a motor problem [3, 12]. In this context, studies have explored cognitive impairments as a risk factor for falls in PwMS [13–15]. The prevalence of cognitive impairments in persons with MS is high, with estimates ranging from 43 to 65% [16]. Processing speed, attention, executive functioning (EF), learning, and memory are common areas of cognition affected by MS [17, 18].

The effect of cognitive deficits on risk of falls in PwMS has been explored via several research paradigms. Firstly, cross-sectional epidemiological studies have employed general measures of cognitive function, such as the Minimental State Examination to determine its relationship with falls through correlation analysis and/or regression models [1–3, 8, 14, 15, 19]. Cognitive capacities of sustained attention, processing speed, and executive functioning appear to be related to falls in PwMS. Nevertheless, the literature does not address whether successful cognitive remediation reduces risk of falling or impacts mobility limitations.

A different experimental design is based on dual tasking, describing the ability to carry out more than one task at the same time. If cognitive resources are limited in capacity and if both gait and a secondary task are attention demanding, performance of at least one of the tasks will deteriorate when simultaneously performed [20]. Various studies have shown that dual tasking effects are greater among the MS population [13, 14, 21]. However, the evidence regarding its association with falls in PwMS is still controversial. For example, one observational study reported that performance on a Timed Up-and-Go cognitive task was predictive of falls in PwMS [2], whereas another observational investigation reported that changes in walking speed during a ten-meter walk, while engaging in a subtraction task, were not predictive of falls in PwMS [22]. Worth noting is that these studies have several limitations such as absence of a control group matched according to various fall risk factors (e.g., sedentary behavior, psychological status, and medication). Moreover, many studies do not include data relating to baseline performance values of the cognitive task.

Additional weaknesses of previous trials involve the cognitive evaluation process. The majority of studies measured cognitive performance by employing a single cognitive test, a self-reported questionnaire, or general measures of cognition, thus limiting an in-depth view of cognitive capabilities in PwMS. Moreover several tests performed were confounded by prior exposure, age, education, fatigue, and emotional status; hence the achieved score may not solely reflect underlying cognitive impairment [23].

In this context, computerized cognitive testing has the potential to effectively address these limitations. This measurement tool offers several advantages such as greater objectivity and enhanced sensitivity due to precise measurement of response time and frequency of errors. Additionally, the computerized tool has minimal ceiling or floor effects due to adaptive testing designs.

Fear of falling, defined as “a lasting concern about falling that leads to an individual avoiding activities that he/she remains capable of performing” [24], has been linked with falls in the MS population [7]. Activity restrictions due to fear of falling can be devastating to the MS population. Previous studies have demonstrated that reduced physical activity due to falling concerns negatively affects fatigue, spasticity, depression, quality of life, and mobility capabilities in PwMS [25]. Yet, to the best of our knowledge, there is no reported data as to the relationship between level of concern of falling and cognitive measures in PwMS.

Therefore, due to the importance of exploring falls and fear of falls in the MS population, the current study's primary aim was to examine the relationship between definite aspects of cognition measured by a computerized cognitive testing tool on the history of falls in this group. Secondary aims focused on whether cognition performance is correlated to fear of falling, walking velocity, and a patient-rated measure of walking ability. We hypothesized that an association between a history of falling and an elevated fear of falling to cognitive deterioration in PwMS exists.

## 2. Methods

### 2.1. Participants

We retrospectively evaluated data from the Sheba MS's computerized database, a population-based registry documenting demographic and clinical data of all MS patients followed at the Sheba Medical Center, Tel Hashomer, Israel, from January 2012 up to March 2014. The MS Center provides long-term multidisciplinary care and treatment for patients from referral areas all over the country diagnosed with MS and is currently following and treating 3250 patients out of ~5000 MS patients in Israel. Since the establishment of the MS Center, an electronic record-keeping system has been used to archive the patients' demographic, clinical, and imaging data. Inclusion criteria required (1) a neurologist-confirmed diagnosis of definite relapsing-remitting MS according to the revised McDonald criteria [26]; (2) PwMS who had undergone computerized neuropsychological testing; (3) PwMS who had filled out the self-reported MSWS-12, FES-I, and falling status questionnaires; (4) PwMS who had performed a gait lab examination with the GAITRite electronic mat; and (5) cognitive, gait, and self-reported forms measured within a 14-day range. Exclusion criteria included (1) orthopedic disorders that could negatively affect mobility; (2) no history of psychiatric problems; (3) pregnancy; (4) blurred vision; (5) cardiovascular disorders; or (6) taking steroids or fampridine. Participants were divided into groups based on fall history (fallers and nonfallers). A fall was characterized as an event when the participant unintentionally came to rest on the ground or a lower level [1]. Information regarding fall history was collected via a single yes/no question; “Have you fallen during the past year?” The query was answered by the participant together with all self-reported forms. The study was approved by the Sheba Institutional Review Board. All participating subjects signed an informed consent form for the use of their data in research projects.

### 2.2. Cognition Assessment

All participants completed a computerized cognitive test battery designed to evaluate multiple cognitive domains and detect mild impairment and dementia (Mindstreams, NeuroTrax Corp., NY). Mindstreams employs novel adaptations of traditional neuropsychological tests providing an overall measure of cognitive function as well as evaluation of specific cognitive domains (e.g., memory). Advantages include adaptive testing designs and precise accuracy and reaction time measurements (millisecond level). All tests were run in the same fixed order and all responses were recorded using the mouse or the number pad on the keyboard. Patients were familiarized with these input devices at the beginning of the test battery and were provided with practice sessions prior to each test with instructions as to the particular responses required. Outcome measurements included 65 parameters from 10 tests covering the following cognitive domains: verbal and nonverbal memory, executive function, visual spatial processing, verbal function, attention, information processing speed, and motor skills. To facilitate the summarization of the performance in each cognitive domain across different types of outcome parameters (e.g., accuracy, RT), each outcome parameter was normalized and fit to an IQ-like scale (mean: 100, SD: 15) in an age- and education-specific fashion. These seven index scores served as the primary dependent variables for the present analysis. A Global Cognitive Score (GCS) computed as the average of these index scores served as a secondary dependent measure. A detailed description of the test battery, index scores, test description, and the outcome parameters are demonstrated in Table 1.

Cognitive scores from this tool have been found to have good test-retest reliability and construct validity relative to paper-based tests in the MS population [27, 28]. Specifically, in PwMS, cognitive scores from this tool showed discriminate validity for memory, information processing, executive function, attention, and motor skill domains [29]. Recently, Mindstreams was used to determine rates and patterns of cognitive impairment in a large cohort of PwMS (*n* = 1500) [30]. An occupational therapist specialized in neurological rehabilitation managed the familiarization phase and provided guidance allied with the computerized cognitive test. Completion of the cognitive tests was approximately 40 minutes. All tests were performed at the Multiple Sclerosis Center, Sheba Medical Center.

### 2.3. Fear of Falls Measurement Tool

The patient's self-reported questionnaire, Falls Efficacy Scale International (FES-I), was used to assess the level of concern relating to falls during 16 activities of daily living, ranging from basic to more demanding activities, including social activities that may contribute to quality of life. Level of concern for each item was scored on a four-point scale (1 = not at all concerned, 4 = very concerned) within a total score range of 16–64; the higher the score, the more the fear of falling. The FES-I was originally designed to assess concern relating to falls in the elderly [21, 22]. Van Vliet et al. showed that the FES-I provides valuable information relating to the fear of falling in PwMS [31].

### 2.4. Walking Assessments

Walking speed was determined by the GAITRite system (CIR systems, Havertown, PA, USA), consisting of a 4.6 m long electronic walkway containing 2304 compression-sensitive sensors arranged in a grid pattern. As the subject ambulated across the walkway, pressure exerted by the feet activated the sensors. Simultaneously, targeted software utilized special algorithms to automatically group the activated sensors and form footprints. The system integrated all footprints and provided spatiotemporal parameters of gait. All participants were instructed to walk barefoot along the mat at a self-selected speed. Gait speed was reported as a mean of six trials.

The Multiple Sclerosis Walking Scale (MSWS-12) is a patient-rated measure of walking ability [32]. The questions were based on the patient's walking limitations due to MS during the past 2 weeks. Each item is scored on a 1 to 5 scale. The higher the score is, the more walking difficulties were perceived.

FES-I and MSWS-12 forms were collected by a physical therapist specialized in neurological rehabilitation. Instrumented gait measurements were performed at the Center of Advanced Technologies in Rehabilitation Center, Sheba Medical Center, Tel Hashomer, Israel.

### 2.5. Statistical Analysis

Group differences in age and gender distribution were determined using an independent sample *t*-test and chi-square test, respectively. All cognitive data were normally distributed according to the Kolmogorov-Smirnov test. Thus, differences in dependent variables between fallers and nonfallers groups were determined utilizing two-tailed independent samples *t*-tests. The magnitudes of group differences were indexed by a 95% confidence interval (95% CI).

Spearman's rho correlation coefficient tests were performed to examine the associations between fear of falling represented by the FES-I questionnaire and gait velocity, patient-based measure of walking ability represented by the MSWS-12 questionnaire, and computerized cognitive parameters.

Evaluation of potential predictors for fall status (i.e., the dependent variable) computing the odds ratio (OR) with their relative 95% CIs by a forward stepwise multivariate logistic regression analysis was performed including covariates: EDSS, gender, age, disease duration, MSWS-12, FES-I, gait velocity, and cognitive variables. At each subsequent step, the regression calculations detect those variables reaching specific thresholds of *F* and *P* values (for variable inclusion, *F* ≥ 1 and *P* ≤ 0.05; for exclusion, *F* < 1 and *P* > 0.05). In order to eliminate redundancy between correlated cognitive variables, a principal component analysis (PCA) was executed prior to the regression model. This procedure extracted variables with the most variance, thus limiting a type I error. The PCA included the Kaiser-Meyer-Olkin measure of sampling adequacy and Bartlett's test of sphericity. Extraction of variables was based on eigenvalues >1.0. Varimax normalization was used as the rotation method.

All analyses were performed using the IBM SPSS statistics software (Version 21.0 for Windows, SPSS Inc., NY, USA). All reported *P* values were two-tailed. The level of significance was set at *P* < 0.05.

## 3. Results

### 3.1. Participants' Demographics

One hundred and one relapsing-remitting patients diagnosed with MS, 60 women and 41 men, aged 40.2 (S.D = 11.9) met the criteria and were included in the study analysis. As a combined group, participants had been ill with MS for an average of 5.4 years (S.D = 6.3). Neurological impairment, as indexed by the neurologist-derived Expanded Disability Status Scale (EDSS) was 3.0 (S.D = 1.8, range 1.5–6) indicating a mild-moderate neurological disability. Fifty-two participants (51.5% of the sample) reported at least one fall during the past year with a further 44 of these fallers (84.6% of fallers) reporting two or more falls during the same period. Forty-nine had no history of falls during the past year. Typically, according to their EDSS score, the individuals within the faller group had a greater level of disability. Moreover, EDSS subscales revealed that fallers had greater impairment in pyramidal, cerebellar, and sensory function than nonfallers. According to the FES-I self-reported questionnaire scores, fallers rated a 71% higher concern of falling compared to nonfallers. Fallers walked significantly slower compared to nonfallers (55.7 (S.E. = 2.5) versus 71.8 (S.E. = 2.5); *P* < 0.001, (m/min)) and reported more walking difficulties according to the MSWS-12; 42.7 (S.E. = 1.9) versus 22.5 (S.E. = 1.8). Demographic and clinical variables of the study population are reported in Table 2.

### 3.2. Cognitive Performance of Fallers versus Nonfallers

Fallers scored less compared to nonfallers. However, significant differences were demonstrated solely in two cognitive domains: attention and verbal function. The mean difference between groups was 6.50 (*P* = 0.046) and 6.74 (*P* = 0.013), respectively. Computerized cognitive scores of the study population are provided in Table 3.

### 3.3. Correlation between Walking and Cognitive Scores

Attention was the only cognitive component significantly correlated with the MSWS-12 self-reported questionnaire (Spearman's rho = −0.277, *P* = 0.005). Gait velocity was significantly correlated with memory scores (Spearman's rho = −0.205, *P* = 0.044) and attention (Spearman's rho = −0.203, *P* = 0.047). Additionally, speed of walking correlated with the global cognitive score (Spearman's rho = −0.239, *P* = 0.018). Correlation scores between the self-reported walking questionnaire and gait velocity to cognitive scores are presented in Table 4.

### 3.4. Correlation between Fear of Falling and Cognitive Scores

Fear of falling was significantly correlated with 6 (out of 7) cognitive variables, in addition to the global cognitive score (Table 4). Hence, poor cognitive performance was related to a greater fear of falling. The strongest correlation was observed in terms of attention, Spearman's rho = −0.364, *P* = 0.004. Other significant correlation scores ranged between 0.2 and 0.3. Visual spatial was the only cognitive variable that did not reach a significance level.

### 3.5. Logistic Regression Analysis Related to Falls

According to step one of the model, MSWS-12 parameter was able to account for 47.4% of the variance related to at least one fall during the past six months; *R* ^2^ = 0.474, *χ*_(1)_^2^ = 37.309, *P* < 0.01. Step two of the model added the gait velocity parameter, thus increasing the explaining variance to 51.7%; *R* ^2^ = 0.517, *χ*_(2)_^2^ = 41.738, *P* < 0.01. Variables not included in the equation were EDSS, FES-I, gender, disease duration, and the cognitive parameters.

## 4. Discussion

In the present study, 51.5% (52 out of 101) of the MS participants reported at least one fall during the past 12 months. The high incidence of accidental falls is similar to previous investigations, ranging from 50% to 63% [1–3], reinforcing the importance of managing fall risk in the MS population.

As a combined group, PwMS demonstrated a reduction in cognitive performance in 7 (out of 7) cognitive domains, in addition to the global cognitive score. In particular, the information processing domain was diminished in PwMS; the mean score was 90.7 (S.E. = 1.6). Although this study did not include a control group, this observation is based on the analysis process of the computerized cognitive software used. Cognitive scores of the MS subjects were standardized and matched according to age and education of healthy people, consequently, scores under 100, representing normal cognitive performance, and indicated reduced cognitive capabilities. Nevertheless, in order to refine the topic of interest, future studies should include a control group of PwMS without a history of falls matched according to other fall risk factors such as sedentary behavior and or nutritional deficiencies.

With regard to cognitive subcategories, the largest decline was observed in information processing, memory, and executive functions. Conversely, visual spatial processing and verbal function were relatively reserved. These findings are in line with previous studies with respect to frequency and pattern of cognitive impairments in the MS population [16].

The primary goal of the study was to examine the relationship between definite aspects of cognition measured by a computerized cognitive testing tool and the history of falls in PwMS. This objective is important considering the magnitude and diversity of cognitive problems in this population [16]. Our findings are controversial, since according to the binary logistic regression analysis, only the MSWS-12 and speed of walking were found to significantly explain the variance related to falling, while the cognitive parameters were nonsignificant. In contrast, we revealed that attention deficits and verbal function were significantly lower in the fallers group compared to nonfallers. Significant differences were not observed in other cognitive subcategories, including the global cognitive score. Moreover, attention deficit was the only cognitive parameter significantly correlated with both gait speed, fear of falling, and self-reported gait disability. Hence, although the strength of the correlation scores was relatively weak (Spearman's rho range 0.2–0.4), we believe that reduced attention is associated with a slower walking pace, low confidence related to falls, and more complaints of walking disabilities.

Attention deficits are less common compared to memory loss. Nevertheless, reduction in attention is known to occur up to 25% in PwMS [16]. Although the exact mechanism underlying the relationship between attention deficits and falls is unclear, as is the sequence of decline in general cognitive and motor functioning, this study is one of the first to reveal these relationships in a relatively large sample of PwMS. According to findings from a five-year prospective study performed on the elderly, it was assumed that a decline in cognitive function may render an individual more prone to distractions while walking and perhaps less competent in the motor-cognitive coordination involved, thus increasing risk of falling [33].

Several magnetic resonance imaging (MRI) studies have evaluated the contribution of white matter hyperintensities (WMHs) and/or the role of focal brain atrophy on both gait and cognitive dysfunction in older adults [31–33]. WMHs were independently associated with gait disturbances and cognitive impairment either irrespective of [34] or together with brain atrophy [35]. In addition, subjects with high-level gait disorders (defined as cautious gait with reduced mobility, significant fear of falling, abnormal postural responses, mild extrapyramidal signs, and frontal release signs) displayed WM changes, as revealed by reduced fractional anisotropy and increased displacement values in regions related to the motor system, as well as in cognitive and affective-related areas [36]. These studies present basic evidence for future studies to examine the relationship between falls, cognitive performance, and changes of various brain regions in the MS population.

In the present study, fear of falling was higher in the MS fallers group compared to nonfallers and significantly correlated with 6 (out of 7) cognitive domains. Participants with low scores on the cognitive tests reported a high concern of falling. Of the various cognitive parameters, the strongest relationship observed was attention deficits (Spearman's rho = −0.364, *P* = 0.001).

To the best of our knowledge, there are no previous studies which examined the association between fear of falling and cognitive impairments in PwMS. In a study performed in the elderly population, lower cognitive scores were correlated to level of fear of falling in 301 community-dwelling older adults (*n* = 301). However, according to multivariate analysis, the authors emphasized that only a high level of depression was associated with fear of falling [37]. Additionally, Brown et al. (2011) confirmed that attentional processing profiles differ between older adults who exhibit fear of falling and those without fear of falling [38]. On the other hand, lower prevalence of fear of falling was associated with memory decline among 101 Japanese older adults [39].

Clinicians are advised to be aware of the negative impact of cognitive deficits on fear of falling. Raising awareness and offering advice from health care professionals may be helpful [40]. Additionally, neuro-psychological-cognitive interventions should also be considered, although the possibility that the fear of falling may be justified and even protective should be considered [41]. In order to reach conclusive evidence as to the relationship between fear of falling and cognitive declines in PwMS, future studies should examine additional risk factors, with an emphasis on psychological components such as depression and anxiety.

From a clinical point of view, the current study provides valuable information. Therapists are advised to carefully examine the impact of cognitive decline, especially attention deficits on falls and concern of falling in PwMS. Although causality between the symptoms cannot be drawn from this study, the negative impact of attention difficulties is relatively clear. Rehabilitation programs aimed at reducing risk and concern of falls are encouraged to examine the effects of different cognitive training strategies on mobility features. Hopefully, evidence from these trials will provide definite data as to formulating a dose-response “receipt” by clarifying important queries such as which mobility parameters are mostly affected by cognitive training or what is the minimal amount of cognitive improvement related to a clinical meaningful change in frequency of falls in PwMS?

The main limitation of this study relates to the measurement of falls. Data was collected in a retrospective manner based on a patient self-reported questionnaire. A possibility exists that participants inadvertently failed to accurately report this incident. Moreover, there is a chance that patients did not want to report falling due to guilt feelings. Additionally, we were unable to determine whether cognitive impairments preceded motor problems or motor dysfunction preceded cognitive problems, or if they occurred concurrently. Therefore, a confirmation prospective study with new MS patients from different geographical locations would help in determining the success of the cognitive parameters, especially attention deficits, in order to predict falls in PwMS. Furthermore, falls may be due to the sum of multiple impairments (i.e., fatigue and spasticity), which were not included in the current study.

## 5. Conclusion

The present findings support the theory that when evaluating and attempting to reduce fall risk and fear of falling, emphasis should be placed not only on traditional risk factors such as muscle strength and motor function, but also on cognitive function. Findings of the current study may be beneficial for follow-up studies. A prospective study designed to examine the degree to which specific cognitive deficits lie in the causal pathway of falls and fear of falling would be especially informative. Moreover, clarifying the temporal and the likely causative relationships between cognitive and gait impairments (e.g., first gait changes or cognitive dysfunction) could aid in the early identification of PwMS who have an increased risk for falls or cognitive impairments.

## Figures and Tables

**Table 1:** MindStreams global assessment battery (GAB): description of cognitive domains tested and outcome parameters obtained.

GAB test	Cognitive domains tested	Test description	Outcome parameters
Go-NoGo response inhibition	Executive function, attention	Timed continuous performance test during which responses are made to large colored stimuli that are any color but red.	Accuracy Average response time Response time standard deviation Errors of commission Errors of omission Response time for errors of commission

Verbal memory	Memory	Ten pairs of words (the study set) are presented, followed by a recognition test in which one member (the target) of a previously presented pair appears together with a list of four candidates for the other member of the pair. There are four immediate repetitions and one delayed repetition after 10 minutes.	Immediate recognition Accuracy, repetition 1 to 4 Accuracy, repetitions 1–4 Delayed recognition Accuracy

Nonverbal memory	Memory	This is similar to the verbal memory test, except that geometric figures are used instead of words.	Immediate recognition Accuracy, repetition 1 to 4 Accuracy, repetitions 1–4 Delayed recognition Accuracy

Problem solving	Executive function	Pictorial puzzles of gradually increasing difficulty are presented. Each puzzle consists of a 262 array containing three black-and-white geometric forms with a certain spatial relationship among them and a missing form. Participants must choose the best fit for the fourth (missing) form from among six possible alternatives.	Accuracy (nonverbal IQ)

Stroop interference	Executive function, attention	Timed test of response inhibition and set shifting. For example, in the “No Interference [Meaning]” phase, the task is to choose the color named by a word presented in a white letter-color. In the final (“Interference”) phase, participants choose the letter-color of a word that names a different color.	No interference: letter color accuracy Average response time Response time standard deviation No interference: word meaning accuracy Average response time Response time standard deviation Interference: letter color versus word Meaning Accuracy Average response time Response time standard deviation

Finger tapping	Motor skills	Participants must tap on the mouse button with their dominant hand.	Inter-Tap Interval Tap Interval Standard Deviation

Catch game	Executive function, motor skills	A test of motor planning requiring hand-eye coordination and rapid responses. Subjects ‘‘catch” a ‘‘falling object” by moving a ‘‘paddle” horizontally on the computer screen so that it can be positioned directly in the path of the falling object.	Time to make 1st move Time to make 1st move standard deviation Average direction changes per trial Average error for missed catches Total score

Information processing speed	Attention, information processing speed	This test comprises three levels of an information processing load: single digits, two-digit arithmetic problems (e.g., 5 − 1), and three-digit arithmetic problems (e.g., 3 + 2 − 1). For each of the three levels, stimuli are presented at three different fixed rates, incrementally increasing as testing continues. Participants are instructed to respond as quickly as possible by pressing the left mouse button if the digit or result is less than or equal to 4 and the right mouse button if it is greater than 4.	SINGLE DIGIT Slow speed, medium speed, fast speed Accuracy Average response time Response time standard deviation TWO-DIGIT ARITHMETIC Slow speed, medium speed, fast speed Accuracy Average response time Response time standard deviation THREE-DIGIT ARITHMETIC Slow speed, medium speed, fast speed Accuracy Average response time Response time standard deviation

Verbal function	Verbal function	Pictures of common objects are presented; in the first phase, the word that best rhymes with the name of the object must be selected from among four choices; in the second phase, the name of the picture must be selected.	Rhyming Accuracy, high and low familiarity Naming Accuracy, high and low familiarity

Visual spatial processing	Visual spatial	Computer-generated scenes containing a red pillar are presented. Participants must select the view of the scene from the vantage point of the red pillar.	Accuracy

Index scores explanation for GAB cognitive tests.

(1) MEMORY: mean accuracies for learning and delayed recognition phases of Verbal and NonVerbal Memory tests.

(2) EXECUTIVE FUNCTION: composite scores (accuracy divided by average response time) for interference phase of the Stroop test and Go-NoGo test, mean weighted accuracy for catch game.

(3) VISUAL SPATIAL: mean accuracy for Visual Spatial Processing test.

(4) VERBAL FUNCTION: weighted accuracy for verbal rhyming test (part of Verbal Function test).

(5) ATTENTION: mean response times for the Go-NoGo test and a no interference phase of the Stroop test, mean response time for a low-load stage of Information Processing test, mean standard deviation of response time for the Go-NoGo test, and mean accuracy for a medium-load stage of Information Processing test.

(6) INFORMATION PROCESSING: composite scores (accuracy divided by average response time) for various low- and medium-load stages of the Information Processing test.

(7) MOTOR SKILLS: mean time until first move for catch game, mean intertap interval, and standard deviation of intertap interval for Finger Tapping test.

**Table 2:** Demographics and clinical characteristics of the study group.

Variable	Mean (S.E.)		*P* value
	Fallers (*n* = 52)	Nonfallers (*n* = 49)	
Age (years)	43.2 (1.7)	38.8 (1.7)	0.07
Gender			
Male	23	19	—
Female	29	30	—
Ratio (F/M)	0.79	0.63	—
Disease duration (years)	6.8 (1.0)	4.6 (0.8)	0.09
EDSS	3.6 (0.2)	2.2 (0.3)	<0.001
Pyramidal	2.3 (0.2)	1.5 (0.2)	0.02
Cerebellar	1.4 (0.2)	0.7 (0.1)	<0.001
Sensory	1.1 (0.1)	0.5 (0.1)	<0.001

FES-I	36.6 (1.6)	21.4 (1.1)	<0.001
Gait velocity (m/min)	55.7 (2.5)	71.8 (2.5)	<0.001
MSWS-12	42.7 (1.9)	22.5 (1.8)	<0.001

EDSS: expanded disability status scale; FES-I: Falls Efficacy Scale International; MSWS-12: Multiple Sclerosis Walking scale.

**Table 3:** Cognitive scores of study population.

Cognitive variable	Mean (S.E.)			Mean difference (95% CI)	*P* value
	Total group (*n* = 101)	Fallers (*n* = 52)	Nonfallers (*n* = 49)		
Memory	**92.1 (1.7)**	90.2 (2.4)	93.9 (2.4)	−3.71 (−10.51, 3.08)	0.281
Executive function	**93.0 (1.2)**	92.7 (1.8)	93.4 (1.6)	−0.69 (−5.53, 4.11)	0.777
Visual spatial	**97.4 (1.7)**	97.1 (2.2)	97.6 (2.7)	−0.49 (−7.37, 6.43)	0.888
Verbal function	**97.5 (1.7)**	94.3 (2.9)	100.8 (1.4)	−6.50 (−13.00, 0.01)	0.046*
Attention	**94.3 (1.4)**	91.1 (2.3)	97.8 (1.3)	−6.74 (−12.03, −1.45)	0.013*
Information processing	**90.7 (1.6)**	88.9 (2.5)	92.5 (2.1)	−3.54 (−10.07, 2.97)	0.283
Motor skills	**94.9 (1.3)**	93.8 (2.1)	96.1 (1.5)	−2.28 (−7.46, 2.80)	0.374
Global cognitive score	**93.2 (1.3)**	**91.8 (1.7)**	**94.6 (2.0)**	**−2.78 (−7.96, 2.42)**	**0.290**

*P-Value < 0.05.

Each outcome parameter is normalized and fit to an IQ-like scale (mean: 100, SD: 15) stratified by age and education.

**Table 4:** Pearson's *r* correlation (*P* values) scores between cognitive variables to walking measurements and fear of falls.

Cognitive variable	MSWS-12	Gait velocity	FES-I
Memory	−0.186 (0.060)	0.205* (0.044)	−0.281** (0.004)
Executive function	−0.150 (0.133)	0.096 (0.350)	−0.243* (0.014)
Visual spatial	−0.107 (0.282)	0.139 (0.173)	−0.119 (0.231)
Verbal function	−0.183 (0.076)	0.079 (0.456)	−0.296** (0.004)
Attention	−0.277* (0.005)	0.203* (0.047)	−0.364** (0.001)
Information processing	−0.202 (0.051)	0.076 (0.477)	−0.231* (0.025)
Motor skills	−0.216* (0.031)	0.115 (0.272)	−0.254* (0.011)
Global cognitive score	**−**0.170** (0.084)**	0.239* (0.018)	−0.248* (0.011)

*P < 0.005; **P < 0.001.

MSWS-12: Multiple Sclerosis Walking Scale-12; FES-I: Fall Efficacy Scale International.
